# Statistical inference of a convergent antibody repertoire response to influenza vaccine

**DOI:** 10.1186/s13073-016-0314-z

**Published:** 2016-06-03

**Authors:** Nicolas B. Strauli, Ryan D. Hernandez

**Affiliations:** Biomedical Sciences Graduate Program, University of California, San Francisco, CA USA; Department of Bioengineering and Therapeutic Sciences, University of California, Byers Hall, 1700 4th Street, San Francisco, CA 94158 USA; Institute for Human Genetics, University of California, San Francisco, CA USA; Institute for Quantitative Biosciences (QB3), University of California, San Francisco, CA USA

## Abstract

**Background:**

Vaccines dramatically affect an individual’s adaptive immune system and thus provide an excellent means to study human immunity. Upon vaccination, the B cells that express antibodies (Abs) that happen to bind the vaccine are stimulated to proliferate and undergo mutagenesis at their Ab locus. This process may alter the composition of B cell lineages within an individual, which are known collectively as the antibody repertoire (AbR). Antibodies are also highly expressed in whole blood, potentially enabling RNA sequencing (RNA-seq) technologies to query this diversity. Less is known about the diversity of AbR responses across individuals to a given vaccine and if individuals tend to yield a similar response to the same antigenic stimulus.

**Methods:**

Here we implement a bioinformatic pipeline that extracts the AbR information from a time-series RNA-seq dataset of five patients who were administered a seasonal trivalent influenza vaccine (TIV). We harness the detailed time-series nature of this dataset and use methods based in functional data analysis (FDA) to identify the Abs that respond to the vaccine. We then design and implement rigorous statistical tests in order to ask whether or not these patients exhibit a convergent AbR response to the same TIV.

**Results:**

We find that high-resolution time-series data can be used to help identify the Abs that respond to an antigenic stimulus and that this response can exhibit a convergent nature across patients inoculated with the same vaccine. However, correlations in AbR diversity among individuals prior to inoculation can confound inference of a convergent signal unless it is taken into account.

**Conclusions:**

We developed a framework to identify the elements of an AbR that respond to an antigen. This information could be used to understand the diversity of different immune responses in different individuals, as well as to gauge the effectiveness of the immune response to a given stimulus within an individual. We also present a framework for testing a convergent hypothesis between AbRs; a hypothesis that is more difficult to test than previously appreciated. Our discovery of a convergent signal suggests that similar epitopes do select for antibodies with similar sequence characteristics.

**Electronic supplementary material:**

The online version of this article (doi:10.1186/s13073-016-0314-z) contains supplementary material, which is available to authorized users.

## Background

Since the administration of the first designed vaccine by Edward Jenner in 1796 [1], vaccines have proven indispensable for both medicine and medical research. Jenner’s work on vaccines are among the rare achievements of science that have fundamentally changed modern life. Perhaps less well-known, vaccines also provide a standardized, safe, and ethical way to directly study human adaptive immunity [2]. Most vaccines confer resistance to a given pathogen by stimulating the patient’s population of B cells to produce antibodies (Abs) against the inoculated antigens. Each clonal lineage is composed of B cells that are related by a single common naïve B cell ancestor and the conglomerate of B cells within an individual make up their antibody repertoire (AbR).

Interestingly, the process by which Abs are adapted to more specifically target an insulting antigen is an example of evolution by natural selection. To wit, during B cell development a vast amount of genetic diversity is generated by a series of somatic mutagenic steps, after which variants that are able to bind an antigen strongly will be positively selected to proliferate [3, 4]. The first diversity-generating step in B cell development is a process of somatic recombination that takes place in the bone marrow. The mature Ab protein is composed of two identical light chains and two identical heavy chains. A light chain can be of either the lambda (IGL) or kappa (IGK) variety, whereas the heavy chain has only one possibility (IGH), and the loci encoding these three chains reside in distinct regions of the genome. Here, the Variable (V), Diversity (D), and Joining (J) gene segments in the IGH locus, and V and J gene segments in the light chain loci will recombine [5–7]. Diversity is generated both by selecting one combination out of all the possible combinations of V, D, and J genes, as well as by the random insertion and deletion of genetic information at the junctions of these gene segments [8]. Further, once a mature B cell binds an antigen, it will be recruited to a lymph follicle and enter a structure known as the germinal center where a process of somatic hyper-mutation (SHM) takes place [3, 4]. Random point mutations are smattered onto the variable region of the Ab locus—the area that is responsible for binding antigen—and if these mutations result in high binding affinity, the B cell clone will receive signals to proliferate. This process generates lineages of B cells specific for a given antigen. These mutagenic steps together result in a high concentration of mutations occurring in a region of the Ab called the complementary determining region 3 (CDR3), which happens to be the region of the Ab that tends to physically interact with antigen. Because of this, the sequence encoding the CDR3 is often used for clonal analysis of Abs, where Abs with the same CDR3 sequence are assumed to be clones. The net effect of this evolutionary process produces extreme temporal dynamism within the AbR, as different lineages grow and shrink in response to different antigenic stimuli [9].

Advancements in next-generation sequencing (NGS) methods have led to recent work in characterizing the AbR’s response to a variety of stimuli [9–21] (see Galson et al. [2] for a review). However, most of this work has focused on methods development, and there has been comparatively little work focusing on what can actually be learned from these data. Contrary to this trend, Greiff et al. [22] recently employed a machine learning approach to classify patients’ immune status using their AbR sequence data. Much work remains to be done in this relatively new area of research. For example, the overall changes in a patient’s AbR could be used to quantitatively assess the response to vaccination. Of particular interest is the ability to use changes in the frequency of individual Abs over time to identify which specific monoclonal Abs (mAbs) respond to a given antigen [23]. For example, if a particular Ab mRNA sequence exhibits a spike in expression in a time-series RNA-seq dataset from peripheral blood, then this could be indicative of a vaccine response for that Ab. To address this gap in knowledge, we here seek to leverage time-series information of five patients’ AbRs in order to infer the elements that are responding to a trivalent influenza vaccine (TIV).

A particularly useful and intuitive way to model time-series data is to use methods within the greater discipline of functional data analysis (FDA) [24, 25]. As opposed to multivariate data analysis (MDA)—which treats each datum as a finite dimensional vector of observations—FDA treats each datum as a continuous function over some dimension, which is often (as in our case) time. FDA-based methods have a rich history of being used for identifying differentially expressed genes over time [26–29], and have the advantage of easily incorporating uneven time-point sampling and measurement error into each gene’s functional model. FDA is also an intuitive way to model gene expression, as each gene’s expression level in a tissue is indeed continuously fluctuating over continuous time. Here, we use an FDA-based method presented by Wu and Wu [28] and apply it to time-series AbR data [30] to identify the components of patients’ AbR that respond to a standard TIV.

There is a plethora of time-series gene expression data that have been used to identify genes involved in pathogen defense [31], autoimmunity [31], and vaccine response [30, 32]. The longitudinal and cross-sectional nature of these studies allowed the authors to identify the genes that were consistently differentially expressed in response to the given antigenic stimulus across patients. One could perform a similar analysis using a time-series AbR dataset to help identify the determinants of immunity. However, with the exception of Liao et al. [33], Laserson et al. [9], and more recently Hoehn et al. [34], few detailed time-series datasets on the AbR exist. If RNA-seq was performed on an antibody expressing tissue (for example, peripheral blood mononuclear cells [PBMCs]), theoretically, many of the RNA transcripts in the data would originate from Ab loci. Should this be the case, much AbR information will exist within the data that simply needs to be bioinformatically mined out. This approach has been used in the context of cancer research to identify the Ab sequence of the cancerous B cell lineage in chronic lymphocytic leukemia patients [35] and to characterize both the AbR and T cell receptor diversity in solid tumor samples [36, 37]. In this study, we developed and implemented such a pipeline on the Henn et al. 2013 transcriptomic dataset [30] in order to probe the AbR’s response to a standard TIV.

There have been several reports of convergent evolutionary signals between independent AbRs that were exposed to a similar antigenic stimulus (recently reviewed by [38]). While there exist relatively precise definitions for convergence in evolutionary biology [39, 40], we define convergent AbRs more loosely as those that develop similar characteristics as a response to similar antigens. These characteristics could include similar Ab DNA sequences, similar sets of Ab genes, or similar structural characteristics, among others. In this manuscript, we focus on convergence by way of independent AbRs utilizing similar sets of Ab genes and similar sequences of CDR3s to target the same vaccine. AbR convergence has been shown in a variety of contexts, including dengue virus infections [21], broadly neutralizing Abs against Human Immunodeficiency Virus [15, 18] and influenza vaccination [23, 41, 42]. With the exception of Parameswaran et al. [21] and Cortina-Ceballos et al. [23], these studies relied largely on qualitative evidence for convergence, where Ab sequences from independent patients either cluster closely together on a dendogram [15] or have strikingly similar sequence and/or structural characteristics [18, 41, 42]. While these examples of AbR convergence may be intuitively convincing, few methods have been developed to statistically test for a convergent AbR response across patients. The importance of statistical analyses can be illustrated by the high correlation of Ab gene expression in different individuals [43, 44]. That is, if an Ab gene is expressed highly in one individual, it will tend to also be highly expressed in another individual. In order to soundly establish a convergent signal between patients’ AbRs, this correlation in background gene expression must be taken into account. Indeed, Childs et al. [45] have used a computational modeling approach to show that a large determinant of AbR diversity post inoculation is its diversity state prior to inoculation. To resolve this, we developed and implemented a statistical methodology that incorporates the baseline similarity between individual AbRs when testing for a convergent signal.

In this study, we first present a bioinformatic pipeline for extracting AbR information from RNA-seq data. We then go on to use FDA-based methods to characterize the Ab response of several patients to a standard TIV. Finally, we present and implement statistical tests for a convergent Ab response between patients to the same TIV. We find that a detailed time-series dataset can be used to identify Abs that are putatively targeting a vaccine, and that—after controlling for background AbR similarities—these vaccine responding Abs can exhibit similar sequence characteristics across patients.

## Methods

### Data creation

The RNA-seq dataset for this study was generated by Henn et al. [30] [GEO:GSE45764] [46]. The experimental design was as follows: five patients were vaccinated with the 2010 seasonal TIV and peripheral blood was drawn from each patient for 11 days, from day 0 (the day of the vaccination) to day 10 post vaccination. Each patient/time-point sample was divided into two sample types: PBMCs and sorted B cells. RNA-seq was performed on both the PBMC and B cell sample types from each time point for all patients. Importantly, the two different sample types from each sample provide relatively independent technical replicates to gauge the accuracy of our bioinformatic pipeline, described below.

For a detailed description of sample processing and RNA-seq, see [30]. Briefly, PBMCs were isolated using a discontinuous Ficoll gradient centrifugation, and B cells were enriched from heparinized whole blood with RosetteSep Immunodensity separation (Stemcell Technologies, Vancouver, BC, Canada). RNA was extracted with the Qiagen RNeasy micro kit. Barcodes were assigned to each patient/time-point/sample type and sequencing libraries were prepared with Illumina TruSeq RNA kits as recommended by Illumina, using 100 ng total RNA as input. The read length was 65 bases and the mean read depth across patient/time-point/sample types was 13,724,354.04 reads, with a range of 8,262,317–17,777,695 reads.

### Computational pipeline

**Fig. 1:**
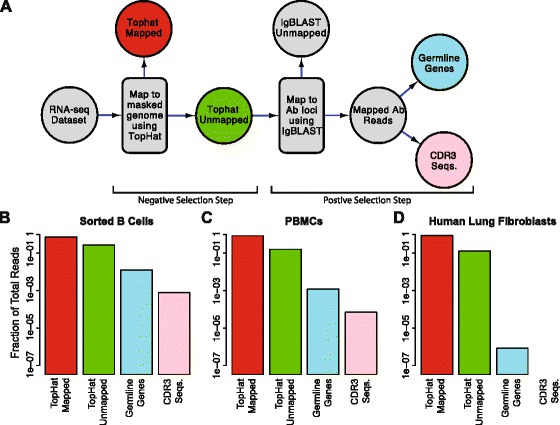
Bioinformatic pipeline. **a** *Flow diagram* of the steps in our bioinformatic pipeline for harvesting Ab reads from a RNA-seq dataset. The pipeline consists of a negative selection step using TopHat2 [47] where non-Ab reads are mapped to a masked reference genome, followed by a positive selection step using IgBLAST [48] where Ab reads are mapped to reference germline Ab sequences. **b-d ** Fraction of reads retrieved for certain steps in the pipeline, in three different tissues, out of the number of TopHat mapped reads (*red*). The colors of the bars correspond to the colors of the steps in (**a**). **b** Sorted B cells from peripheral blood. **c** Peripheral blood mononuclear cells. **d** Human lung fibroblasts from tissue culture

State-of-the-art tools for aligning RNA-seq reads to a reference genome, such as TopHat2 [47], were not designed, and are ill-equipped, to handle the various eccentricities of Ab RNA (such as VDJ gene segment recombination, as well as the high number of mutations expected from both VDJ recombination and SHM). Similar to others [35, 36], we therefore developed a bioinformatic pipeline that will harvest the Ab transcripts buried in the multitude of reads from an RNA-seq dataset. Conceptually, the pipeline consists of a negative selection step to weed out all non-Ab encoding transcripts, followed by a positive selection step to identify Ab encoding reads (Fig. 1a). For the negative selection step, we first created a whole genome reference sequence where all Ab encoding loci in the genome were masked out. We then used TopHat2 to map all reads to this masked-reference genome. Reads that successfully mapped to the masked genome were discarded. We hypothesized that some fraction of the unmapped reads are true Ab sequences. To identify them, we used IgBLAST [48] to positively select for Ab encoding transcripts. We used a stringent threshold (e-value ≤ 10^−20^) to select the best aligning germline Ab gene (including V, D, and J genes). We also selected the CDR3 sequence in the alignment (if present) using a less stringent e-value threshold of 10^−6^ in order to retrieve a sufficient number of CDR3 sequences.

### Overall Ab expression and V gene expression

We would like to measure the overall level of mRNA expression of Abs, as well as the expression level of individual V genes and CDR3 sequences in each sample. By ‘expression’ we mean a quantitation of the number of mRNA sequences that map to a given Ab locus in this peripheral blood RNA-seq dataset. We first would like to estimate the overall expression of Abs in each sample. To do this, we counted the number of mRNA reads that mapped to any gene (V, D, or J) in the variable regions of any Ab locus (heavy, lambda, or kappa chains), and then normalized this by the number of reads that map to anything else in a given sample. We will henceforth refer to this statistic as “overall Ab expression,” and it was calculated as follows. Let *T* be the total number of days in the study, with *t* ∈ [0, *T*], and *P* be the total number of patients, with *i* ∈ [1, *P*]. For a given patient *i* and time-point *t*, if *M*_*i*,*t*_ is the total number of reads that map to a V, D, or J gene with an *e*-value ≤ 10^−20^, and *N*_*i*,*t*_ is the total number of reads that map to anything in the Ab-masked genome (the red circle in Fig. 1a), then overall Ab expression, *A*_*i*,*t*_, for that patient/time-point can be calculated as

$$ {A}_{i,t}=\frac{M_{i,t}}{N_{i,t}+{M}_{i,t}}. $$

Because *M*_*i*,*t*_ was very small rative to *N*_*i*,*t*_, we approximated overall Ab expression as

$$ {A}_{i,t}={M}_{i,t}/{N}_{i,t}. $$

It is important to note that we do not attempt to map reads to any of the constant regions of the Ab loci (IgA, IgE, IgM, IgG, etc.), so our expression level estimates are agnostic to this information. As such, overall Ab expression is a measure of the cumulative mRNA expression of all isotypes in a sample.

Next, we would like to estimate the mRNA expression level for each individual Ab gene. We achieved this by counting the total number of reads that mapped to a given Ab gene, then normalized by both the number of reads that mapped to anything else (as was done for overall Ab expression), as well as by the length of the Ab gene. We will hereto refer to this statistic as “gene expression” and it was calculated as follows. Let *V* be the total number of unique genes that we detected belonging to a given Ab gene class (e.g. for IGHV, *V* = 68; excluding alleles). For *v* ∈ [1, *V*], let *L*_*v*_ be the length of gene *v*. If *m*_*v*,*i*,*t*_ is the total number of reads that map to Ab gene *v* with an e-value ≤ 10^−20^, then the gene expression level, *E*_*v*,*i*,*t*_, of Ab gene *v*, in patient *i* at time-point *t* was calculated as

$$ {E}_{v,i,t}=\frac{m_{v,i,t}}{N_{i,t}{L}_v} \cdot 1000. $$

Lastly, we would like to estimate the mRNA expression level of a given CDR3 sequence. This statistic is referred to as “CDR3 expression,” and was calculated by counting the number of times the CDR3 sequence was observed in patient *i* at time-point *t*, normalized by *N*_*i*,*t*_.

### Ab diversity

We used the CDR3 sequences in our dataset to estimate AbR diversity. We calculated the mean pairwise genetic distance (commonly referred to as *π* in population genetics) as our diversity statistic. However, there were different numbers of total reads sequenced for each patient/time-point and comparing diversity estimates across differing sample sizes is problematic, as the variance of the estimate can change dramatically. To account for this, we down-sampled our data until the number of reads for each patient/time-point was equal to the time-point with the least reads. We then calculated diversity from this down-sampled data. To account for possible stochastic effects of down-sampling, we analyzed the mean of ten independently down-sampled diversity estimates.

Let *C*_*i*,*t*_ be the total number of unique CDR3 sequences found in patient *i* at time-point *t*, with *c* ∈ [1, *C*_*i*,*t*_]. Let *d*_*i*,*t*,*c*_ be the number of times the CDR3 sequence *c* was found in patient *i* at time-point *t*, with ${U}_{i,t}={\displaystyle \sum_{c=1}^{C_{i,t}}}{d}_{i,t,c}$ being the total number of CDR3 sequences detected. Additionally, let *s*_*i*,*t*_ be a list of inferred CDR3 sequences. Antibody diversity, *π*_*i*,*t*_, for patient *i* at time-point *t* was estimated as

$$ {\pi}_{i,t}=\frac{{\displaystyle {\sum}_{j=1}^{C_{i,t}-1}}{\displaystyle {\sum}_{k=j+1}^{C_{i,t}}}{d}_{i,t,j}\cdot {d}_{i,t,k}\cdot G\left({s}_{i,t,j},\ {s}_{i,t,k}\right)}{\left(\begin{array}{c}\hfill {U}_{i,t}\hfill \\ {}\hfill 2\hfill \end{array}\right)}. $$

Where *G*(*x*, *y*) gives the genetic distance between the two CDR3 sequences, *x* and *y*. This was accomplished using the Needleman-Wunsch algorithm encoded by “needle” in the EMBOSS package to globally align sequences *x* and *y*. We then calculated “genetic distance” by finding the percent of mismatches in this alignment, including gaps.

In words, *π* can be thought of as the genetic distance that would be expected if one were to randomly pull two CDR3 sequences from a population. Thus, if there are many unique CDR3 sequences, yet only a small subset of these sequences have a high frequency, then *π* will be relatively low; conversely, if there are the same set of unique CDR3 sequences but their frequencies are evenly distributed, *π* will be relatively high.

### Comparing B cell and PBMC CDR3 populations

We used a random sampling approach to test whether or not the CDR3 sequences from B cell and PBMC sample types were samples from the same population. Specifically, for a given patient we randomly chose a time-point, then within this time-point, we selected one CDR3 sequence from the B cell dataset and one from the PBMC dataset, where the relative frequency of the CDR3 sequences determined the probability of selection. We then calculated the genetic distance between these two sequences using *G*(*x*, *y*), as was done in the diversity calculation. This process was done 1000 times to create a distribution of genetic distance values. To create null distributions, we repeated this workflow, except sampled pairs of CDR3 sequences from the same population. We used the Mann–Whitney *U* test to determine if the B cell/PBMC distribution of genetic distances was significantly different from either of the nulls. This process was done for each of the patients.

### Test for identifying TIV-responding Abs

The following method was used to identify both TIV-responding V genes and TIV-responding CDR3 sequences, so we shall henceforth use the notation “Ab-element” to refer to either V gene or CDR3 sequence. For a detailed description of this FPCA based test, see Wu and Wu [28] and associated R code [49]. Briefly, the test functions by first converting each of the Ab element’s expression trajectories into a continuous function over the time-course, *t*, which we will call an “expression function.” This is accomplished by finding the linear combination of the naïve basis functions that best fit the observed Ab-element’s expression data. These expression functions, *X*(*t*), can be expressed as

$$ X(t)=\mu (t){\displaystyle \sum_{l=1}^b}{\alpha}_l{\lambda}_l(t). $$

Where *μ*(*t*) is a constant function that is equal to the mean Ab-element expression over the time-course, *α*_*l*_ is the weight given to basis function *λ*_*l*_(*t*), and *b* is the number of basis functions in the model.

FPCA is then performed on this set of expression functions. We then identified the first set of eigenfunctions that explain at least 90 % of the variance in the data. Once this is done, *X* ' (*t*) can be re-expressed as a linear combination of this set of eigenfunctions that best fits the observed data.

$$ {X}^{\hbox{'}}(t) = \mu (t){\displaystyle \sum_{l=1}^c}{\xi}_l{\phi}_l(t), $$

Where *ξ*_*l*_ is the weight for each eigenfunction, *ϕ*_*l*_(*t*), which is often referred to as the functional principal component score, and *c* is the number of eigenfunctions that together explain at least 90 % of the variance in the data (such that their eigenvalues are non-increasing).

Once this is done, the task is then to determine if *X* '(*t*) is a better fit to the data than the null hypothesis. The null in this case is that the Ab-element’s true expression function is *μ*(*t*) (where the observed deviation around the mean is due to random error). Thus, the null hypothesis is *X*^0^(*t*) = *μ*(*t*). It is then determined which of the two hypotheses better fit the data by measuring the residual sum of squares (*RSS*) for the two models, *RSS*' and *RSS*^0^. The test statistic is given by

$$ F=\frac{RS{S}^0-RS{S}^{\hbox{'}}}{RS{S}^{\hbox{'}}+\delta }. $$

Where *δ* is a small constant that is meant to stabilize the variance of *F* and is set to equal the variance of the Ab-elements’ observed expression values around its estimated expression function. Finally, in order to produce a null distribution of the test statistic, a permutation-based approach is used. The time-points are shuffled and this process is repeated. The Ab-elements whose *F* statistics were significant relative to the null distribution were deemed TIV-responding. A Benjamini Hochberg correction for multiple tests was used on the *p* values within a patient/gene class.

### Generation of literature-curated dataset of flu-targeting Abs

In order to characterize the diversity of Abs that have been reported to physically bind influenza, we scanned the literature and recorded the germline gene identity of all influenza-binding Abs that we found. The generation of this literature-curated dataset qualifies as a meta-analysis, so we created a separate Preferred Reporting Items for Systematic Reviews and Meta-Analyses (PRISMA) [50] statement that explicitly addresses each item in the PRISMA checklist in order to clearly outline the criteria used to select the studies that contributed to this meta-analysis. See Additional file 1: PRISMA statement and Additional file 2: PRISMA Flow Diagram in the supplemental information for details on this meta-analysis.

### Test for a globally convergent V gene response

To determine if patients tend to use similar sets of genes to target TIV, we developed a statistic, which we refer to as “sum of gene significances” (SGS) and is defined as the number of patients in which a given gene was found to be significant. Because we have five patients in our data, SGS is bound between 0 and 5. We computed the SGS value for each gene, and then compared the observed SGS distribution to its null. Our task was then to generate a proper null distribution that takes into account the baseline frequencies at which the different V genes are expressed in a given patient, prior to vaccination. We chose to use a simulation-based null model, where we use day 0 gene frequencies to simulate artificial sets of TIV-responding genes.

These null simulations are best explained by example. Say the number of TIV-responding V genes for patients 1 through 5 were: 3, 6, 4, 7, and 4, respectively. The first step is to sample, without replacement, three genes from patient 1’s day 0 distribution of gene frequencies. Here, the probability of sampling a given gene for patient 1 is equal to that gene’s relative frequency at day 0. We then complete the same process in the other patients by sampling six genes from patient 2’s day 0 gene frequency distribution; four genes from that of patient 3; seven genes for patient 4; and four genes for patient 5. We now have “null sets” of V genes from each patient, where the composition of these sets only reflect the gene expression levels prior to vaccination. We can then calculate SGS values for each V gene by counting the number of times each gene is present in a “null set.” For example, if IGHV3-23 was sampled in all patients, then it would have an SGS value of 5, and if IGHV4-59 was sampled in patient 1 and patient 4 then it would have an SGS value of 2. We store these SGS values as a long list of integers. We then repeat the sampling process from each patient’s day 0 gene frequency distribution 1000 times, and after each trial we append the resulting SGS values for each gene to our long list of integers. Once this is done, we can convert this long list of SGS values into a distribution, where this distribution serves as our null, and reflects the SGS values that one might expect to get if they were to randomly sample genes from each patient prior to vaccination. We can then use a multinomial G test to compare our observed SGS distribution to the null.

To generate the “naïve” null distribution, we treated each patient independently and then simulated SGS statistics under this model. We did this by first estimating the probability that a gene will be significant (i.e. deemed TIV-responding) in each of the patients. This was done by dividing the number of V genes found to be TIV-responding in a patient by the total number of V genes found in that patient. Once the probability of significance was estimated in all patients, we simulated SGS values based upon these probabilities. This was accomplished by walking through each patient and randomly assigning them a “1” or a “0” (i.e. significant or non-significant), where the probability of getting a “1” is equal to the probability of significance that was previously estimated for that patient. For example, if 3/10 V genes were found to be TIV-responding in patient 1, then this patient would have a probability of 0.3 (3/10) of being assigned a “1.” This assignment of either “0”s or “1”s was completed for each patient, and by taking the sum across patients we get a simulated SGS value. We then repeat this process 10,000 times to arrive at the distribution of SGS values that one might expect if the probability that a gene is significant in one patient is independent of all the other patients.

### Test for convergent response in individual V genes

This test is similar in spirit to the global test for V gene usage convergence (above), where the day 0 V gene usage is used to generate the null distribution. However, instead of a simulation based approach to generating this null distribution we develop a closed form solution. *P* is again the total number of patients in the study (5 in our case), and *p*_*i*_ is the relative proportion of a given V gene at day 0 in the *i*th patient (where *i* ∈ [1, *P*]). *S* is the set of identifiers for each patient, so *S* = {1, 2, …, *P*}, and *S*_*k*_ is the set of all subsets of *S* that are of size *k*, so *S*_*k*_ = {*x*|*x* ⊂ *S*, |*x*| = *k*}, which represents all the different ways to choose *k* patients from *S*. If *X* is the random variable that describes the number of patients in which a given V gene is significant, then the probability of *X* under the null hypothesis is given by,

$$ \Pr\ \left(X=x\right)={\displaystyle \sum_{y\in {S}_x}}\left[{\displaystyle \prod_{i\in y}}Y\left({p}_i,{g}_i\right){\displaystyle \prod_{j\notin y\mid j\in S}}1-Y\left({p}_j,{g}_j\right)\right]. $$

Where *Y*(*a*, *b*) is a function that gives the probability a gene will be found to be TIV-responding in a single patient, given that that patient has a day 0 gene frequency of *a*, and *b* V genes were observed to be TIV-responding in this individual. *Y*(*a*, *b*) is given as

$$ Y\left(a,b\right)=1-{\left(1-a\right)}^b. $$

Essentially this can be thought of as a traditional urn problem in probability, where each patient is an urn that contains a given proportion of red balls. The probability of selecting a red ball from an urn is the probability of selecting a given V gene from a patient at day 0. The null distribution is modeled as follows: if *g*_*i*_ is the number of draws made from each urn *i* (the number of TIV-responding genes found for patient *i*), and *p*_*i*_ gives the probability of drawing a red ball from urn *i* (the relative frequency of the Ab gene in question at day 0), and *X* describes the number of urns from which red balls are drawn (the number of patients in which a particular V gene is identified as TIV-responding); then the probability of *X* is the null distribution for SGS.

### Power simulations for global V gene convergence test

In order to assess the statistical power of our SGS based tests for convergence, we ran simulations of the data over different parameter values to see how often the simulated data were different than the corresponding null distribution. More specifically, we simulated SGS values for each V gene and our simulations had two parameters that were varied over a range of possibilities. These parameters were: number of truly convergent genes and number of patients in the study. These simulations are best illustrated by example.

Say we wish to run simulations where there are seven patients, and two truly convergent genes. The first step is to create “simulated” patients. Here, since we already have five observed patients, we will only need to create two additional “simulated” patients. For the purposes of the global V gene response convergence test, each patient needs two qualities: a distribution of day 0 gene frequencies and a number of genes that were found to be TIV-responding for that individual. Both of these values are found by randomly selecting from the five existing observed patients. That is, each gene’s day 0 frequency for the simulated patient is found by randomly selecting from the day 0 frequencies for that gene of the five observed patients. All of the randomly selected day 0 gene frequencies in the simulated patient are then re-normalized by their sum to make them relative proportions. The number of TIV-responding genes is also randomly selected from the existing values of the observed patients. This is done independently for each simulated patient. The next step is to simulate convergent genes. Two V genes are randomly selected (regardless of their day 0 frequencies) to be truly “convergent.” This means that they are significant in all patients (i.e. their SGS value is set to equal 7). For each patient, the remainder of V genes are then randomly selected to be TIV-responding based upon their day 0 frequencies, until the number of genes selected for a given patient equals the total number of number of TIV-responding genes for that patient. For example, if patient 1 had five genes that were found to be TIV-responding, then two of these genes are set to be truly convergent (i.e. significant in all patients), and the remaining three are randomly drawn from patient 1’s day 0 distribution of gene frequencies, just as was done for our null distribution. Once this is completed for each patient, we have simulated SGS values for each gene, and thus can arrive at a simulated distribution of SGS values. We then compare this simulated distribution to a null distribution, which is generated the same way as described above, except no “truly convergent” genes are assigned and genes are instead solely sampled based upon their day 0 frequencies. This entire process is then run 10,000 times and power is calculated as the proportion of simulations that yield SGS distributions that are significantly different from the null distribution.

### Power calculations for individual V gene convergence test

We calculated power over a range of parameter values for the convergence test for individual genes. The parameters that we varied for this test were: number of patients in the study and day 0 gene frequency. Because we have a closed form solution for the null distribution of this test, it is not necessary to run simulations, and we can instead calculate power directly from our equation, albeit with a few simplifying assumptions. For this test, each patient needs two qualities: a day 0 gene frequency and number of genes found to be TIV-responding. We assume the day 0 frequency for a gene to be the same across all patients and we set the number of significant genes for each additional patient, beyond the five observed, to be the nearest integer to the mean of the five observed values. We then plug these values into our equation and find the probability that a gene would be found to be significant in all the patients, given a starting frequency and a given number of patients. This provides the probability of the null hypothesis, and we calculate statistical power by subtracting this value from 1.

### Test for convergent CDR3 response

To test if two patients have sets of TIV-responding CDR3s that are more similar to each other than would be expected by chance, we again utilized a methodology that hinges on sampling from the day 0 distribution. First, we calculate *π* (the mean pairwise genetic distance) between the two patients’ observed set of TIV-responding CDR3s. If *X* is the set of TIV-responding CDR3 sequences in patient *x*, and *Y* is that of patient *y*, then *π*_*x*,*y*_ between patient *x* and *y* was calculated as

$$ {\pi}_{x,y}=\frac{{\displaystyle {\sum}_{i\in X}}{\displaystyle {\sum}_{j\in Y}}G\left({X}_i,{Y}_j\right)}{\left|X\left|\cdot \right|Y\right|}. $$

We then generate the null distribution for *π*_*x*,*y*_ by randomly sampling (without replacement) from the population of CDR3 sequences at day 0 for both patients *x* and *y*, where the frequency of each CDR3 sequence determines the probability that it will be sampled. The number of sequences that are sampled for each patient are equal to the number of CDR3 sequences that were found to be TIV-responding for that patient. These sets of CDR3 sequences form a null set and are solely informed by the baseline CDR3 expression level of the sequences prior to vaccination. We then calculate *π*_*x*,*y*_ between the two null sets from patients *x* and *y* and repeat this sampling process 1000 times to get a distribution of null *π*_*x*,*y*_ values. We can then assess significance of an observed *π*_*x*,*y*_ value between two patients by comparing it to the respective null distribution.

### Data and software availability

Data for the immunological assays performed by [30] are available at the ImmPort repository [ImmPort:SDY224],[51]. RNA-seq data generated by [30] are available at the GEO repository [GEO:GSE45764],[46]. The anonymous patients in this study have different naming schemes in different contexts. In this study, patient 1, patient 2, patient 3, patient 4, and patient 5 equates to samples T12, T13, T14, T15, and T16 in the GEO repository; as well as equates to patient IDs S04, S06, S02, S03, and S05 in the Henn et al. study, respectively. All software associated with the analyses herein are available on the GitHub repository (https://github.com/nbstrauli/influenza_vaccination_project) [52].

## Results

In this study, we implemented a pipeline to extract Ab sequences from RNA-seq data in order to take advantage of a unique densely sampled time-series dataset comprising RNA-seq data from PBMCs and sorted B cells of five patients vaccinated with the 2010 seasonal TIV over a time-course of 11 days [30] (Additional file 3: Figure S1). We use the high-resolution temporal information in these data in order to infer the elements of the AbR that are putatively responding to TIV. We then go on to test if the patients in this dataset exhibit more similar responses to TIV than would be expected by chance. That is, we test if these distinct AbRs exhibit convergence in response to the same vaccine.

### Quality control of bioinformatic pipeline

First, we validated that our bioinformatic pipeline (Fig. 1a, see “Methods” for a detailed description) extracts meaningful AbR information from RNA-seq data. We hypothesized that the proportion of Ab encoding reads detected should correlate with the expected number of B cells in a given sample type. We arbitrarily chose the day 7 time-point from patient 1 and applied our pipeline to the RNA-seq data from isolated B cells and PBMCs for this patient/time-point. As a negative control, we also applied our pipeline to RNA-seq data from human tissue cultured lung fibroblasts [53]. Our expectation was that the number of Ab sequences would decrease from B cells to PBMCs and cultured lung fibroblasts would serve as a negative control with essentially no Ab sequences. Consistent with our expectation, we found that 1.25 % of all reads from isolated B cells encode Ab (206,797 of 1.7 × 10^7^ total reads), PBMCs yielded 0.12 % Ab encoding reads (16,214 of 1.4 × 10^7^ total reads), and cultured lung fibroblasts produced < 0.001 % Ab encoding reads (25 of 3.0 × 10^7^ total reads) (Fig. 1b).

### Broad AbR characteristics

**Fig. 2:**
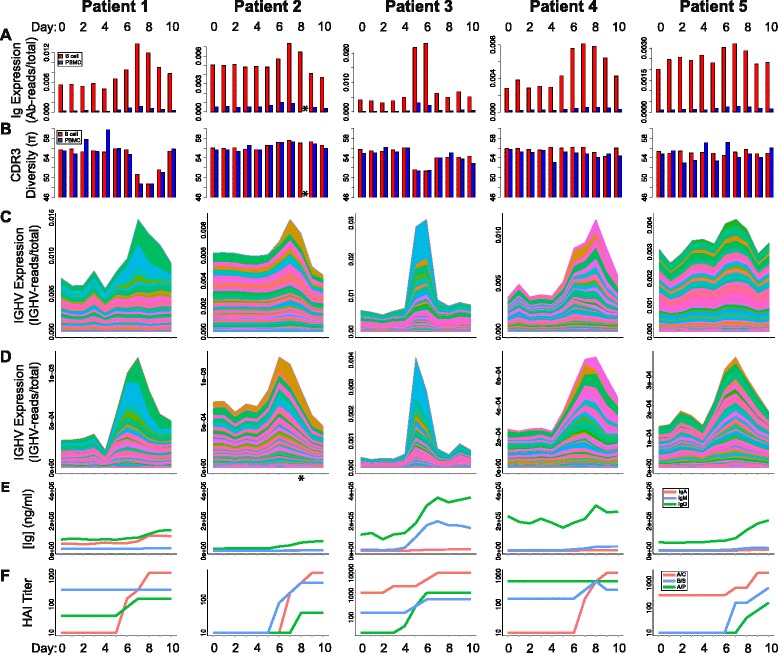
AbR response to TIV across patients. Different metrics were measured for each patient and at each time-point. Metrics are delineated by *row*, and patients are delineated by *column*. **a** Overall Ab expression for each patient/time-point. **b** CDR3 diversity for each patient/time-point. B cells and PBMCs are shown in *red* and *blue*, respectively. **c**, **d** Stacked area charts showing the gene expression level for each IGHV gene for each patient/time-point. Colors, corresponding to IGHV genes, are comparable between patients and sample types, and were sorted by absolute range (max–min). **c** B cell. **d** PBMC data. Complete definitions for the y-axis units in (**a**, **b**, **c**, and **d**) can be found in methods. **e** ELISA results giving the concentration of Abs that bind TIV for the A, M, and G Ab isotypes (*red*, *blue*, and *green*, respectively). **f** Hemagglutinin inhibition assay results for the three different virus strains in the administered TIV, A/C: A/California/7/2009; B/B: B/Brisbane/60/2008; A/P: A/Perth/16/2009. Data for (**e**) and (**f**) were generated by [30], and downloaded from ImmPort [51], [ImmPort:SDY224]. *Patient 2 PBMC data at day 8 were unavailable due to sample processing error [30]

We next sought to characterize how the AbR broadly behaves in response to TIV. To this end we measured overall Ab expression as the number of Ab mapped reads normalized by the number of non-Ab mapped reads, see “Methods” (Fig. 2a). Ab diversity was measured as mean pairwise CDR3 genetic distance (see “Methods”) in each of the patients over the time-course (Fig. 2b). We found that each patient had a characteristic peak in overall Ab expression around day 7, although the timing and severity of this peak varied dramatically across patients. Patient 3 had the most dramatic response, which had entirely subsided by day 7, while the response for patient 5 was much more gradual and less pronounced. We note that patient 3 was the only patient to have received the seasonal influenza vaccine for each of the prior 3 years, and received an additional monovalent vaccine the year prior to the study [30]. The monovalent and seasonal 2009 vaccines had epitopes from two of the strains that were included in the TIV used in this study (Additional file 3: Table S1).

These results are consistent across both the B cell and the PBMC RNA-seq sample types. Indeed, overall Ab expression and diversity levels for each of the patients and time-points are highly correlated between the two sample types (overall Ab expression: Kendall’s tau = 0.639, *p* = 8.715e-12, Ab diversity: Kendall’s tau = 0.366, *p* = 8.083e-05; Additional file 3: Figure S2), suggesting that the overall signal represents the underlying AbR diversity and expression patterns.

### Comparing B cell and PBMC CDR3 populations

Because B cells are a subset of PBMCs, we can expect that the RNA-seq data from these two sample types should yield similar Ab sequences. By checking to see if this is indeed the case in our data, we have another means to check the accuracy of our pipeline. In order to quantify the similarities between the B cell and PBMC sample types, we focused on CDR3 sequence sets. Specifically, we statistically tested whether the CDR3 sequences from the B cell and PBMC datasets are drawn from the same population. We did this by finding the distribution of genetic distances between CDR3 sequences from different sample types and compared this to the same distribution from CDR3s in the same sample type (see “Methods”). We found that none of these three distributions are significantly different in any of the patients (*p* >0.07, see Additional file 3: Figure S3). We thus conclude that PBMC and B cell datasets can reliably be used to extract Ab sequences from RNA-seq data.

### V gene and CDR3 usage analysis

We next sought to analyze how each Ab gene is expressed over the time-course after vaccine administration. We calculated the mRNA expression level of each gene (as number of reads mapped to a given Ab gene normalized by the number of non-Ab mapped reads, see “Methods”) in each of the patients and at each time-point. We analyzed each class of Ab gene that could produce reliable alignments: V gene heavy chain (IGHV), V gene lambda light chain (IGLV), and V gene kappa light chain (IGKV). We were unable to detect an appreciable number of reads aligning to D or J genes with high confidence, which is likely due to their short lengths. We then generated stacked area charts to observe how the cumulative and individual V gene expression changes over time (Fig. 2c, d for IGHV; Additional file 3: Figure S4 for IGLV and IGKV). We find that the patients with the most dramatic Ab response (patients 1 and 3) also seem to have the largest gene expression increases in very few V genes and that the increase in these few genes seem to explain a large portion of their rise in overall Ab expression. This is particularly well illustrated in patient 1, where the peak in overall Ab expression is entirely explained by an increase in gene expression of 2–3 V genes. Moreover, this expression increase coincides with a dip in CDR3 diversity. Together, this suggests that patients 1 and 3 had largely a monoclonal response to TIV. We also note that the other patients showed signs of a predominantly polyclonal Ab response that did not substantially affect diversity. Though we cannot draw strong conclusions about the causes of a polyclonal or monoclonal response in this small sample size, future studies of larger cohorts could elucidate the causes behind this heterogeneity.

We performed an analogous analysis using CDR3 sequence data. We gathered all unique CDR3 sequences for each patient/time-point sample and calculated their mRNA expression level (see “Methods”). We again generated stacked area charts to observe how the predominant sequences change in CDR3 expression over time (Additional file 3: Figure S5). We found that these data largely recapitulate the gene expression data, where CDR3 expression expansions tend to occur around the same time as the increases in V gene expression. Patient 1 again shows a dramatic expansion in the expression of a single CDR3 sequence, providing further support for a largely monoclonal response.

There are two factors that contribute to an Ab’s mRNA expression level: the number of B cells harboring the Ab and the rate of Ab expression for each of these B cells. Because RNA-seq was performed on a heterogeneous population of B cells in the peripheral blood, we cannot distinguish between the two. Further, these two factors are highly dynamic over time, where B cells are constantly migrating in and out of the peripheral blood, in addition to dramatically varying their rate of Ab transcription. Thus, the population of B cells that we sample on day 7 is likely very different from that of day 0. However, whether due to a clonal expansion or an increase in transcription rate, if an Ab gene or CDR3 sequence increases in expression level, it is largely indicative that at least a subset of the B cells harboring this gene or CDR3 are responding to some antigenic stimulus.

### Immunological assays

Given the robust signal in our V gene and CDR3 usage analyses, we sought to validate that the expansions we observed in our data were indeed in response to TIV. Henn et al. [30] performed a variety of immunological assays using the sera from each patient/time-point sample. We downloaded these data from [51], [ImmPort:SDY224] to determine if the patients gain immunological reactivity against influenza around the same time as the V gene and CDR3 expression level expansions occur in our data. The results show that vaccine-binding immunoglobin tended to increase around the same time as V gene and CDR3 expansions (Fig. 2e). We next sought to establish that the V gene and CDR3 response conferred protectivity against influenza virus. Data from hemagglutinin inhibition (HAI) analyses using the three strains of influenza virus included in the TIV showed that protection to at least one of the strains was gained around the same time as the spike in V gene and CDR3 expression levels (Fig. 2f). Together these data suggest that the V gene and CDR3 expression level expansions we observe in our data are direct immunological responses to TIV.

### Identifying TIV-responding V genes

**Fig. 3:**
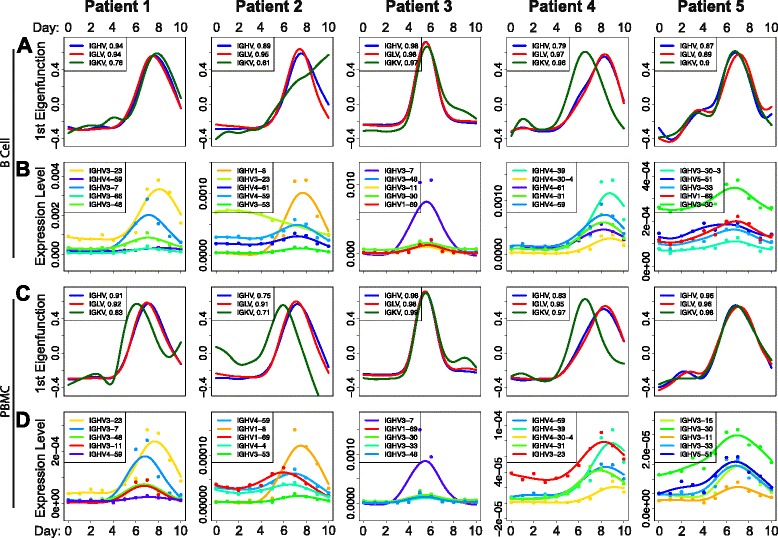
Identifying putative TIV-responding V genes. **a** First eigenfunction in the B cell data for each patient and each V gene class. The proportion of the total variance explained by the first eigenfunction is listed in the legend after each respective class of V gene. **b** The top five scoring IGHV genes from the FPCA based test to identify TIV-responding V genes; in the B cell data. The *points* show the observed data, and the *solid lines* show the best fitting gene expression function over time. V genes in legend are ordered by *p* value, with lowest on top. *P* values are based on a permutation test, see Wu and Wu [28] for details. **c**, **d** Same as (**a**, **b**) but from the PBMC data. *Colors* corresponding to IGHV genes in (**b**, **d**) are comparable within a patient. Eigenfunction *plots* (**a**, **c**) were generated using the “eigens” output from the FPCA-based test. IGHV gene expression functions (**b**, **d**) were plotted using the “fda” package for the R programming language and using a smoothing parameter, “lambda” = 0.66

Given the robust signal that we saw in the V gene expression time-course data, we next established a methodology to systematically identify the V genes that appear to be responding to TIV. We utilized a method based on functional principal component analysis (FPCA), which was designed to identify differentially expressed genes over a time-course [28] (see “Methods” for description). We found that it was often the case that the first eigenfunction explained over 90 % of the variance in the data (Fig. 3a and c). From this method we were able to identify the genes that seem to be most dramatically responding to TIV (Fig. 3b, d; Table 1). In almost all patients, the top genes identified in the B cell dataset (Fig. 3b) are replicated in the PBMC dataset (Fig. 3d). We deem the V genes identified by this test to be “TIV-responding.” It is important to note that while the results of this test provide evidence that these genes are “responding” to TIV, functional validation is required to establish that they actually target TIV. We then assessed whether or not these sets of TIV-responding genes, are more similar across patients then would be expected by chance.

**Table 1:** Top ten TIV-responding heavy chain V genes

Gene name	Lit Ab Freq.	Combined	Patient 1	Patient 2	Patient 3	Patient 4	Patient 5
IGHV3-23	0.10345	1.08E-14	1.54E-05	4.55E-05	0.00529688	0.00010769	0.00015385
IGHV1-69	0.23707	5.04E-14	0.00043284	0.00290909	0.00028125	6.15E-05	1.54E-05
IGHV3-30	0.03879	7.19E-13	0.00153731	0.00154546	0.00025	0.00069231	1.54E-05
IGHV3-7	0.04310	1.15E-12	0.00020896	0.00792424	1.54E-05	0.00010769	0.00389231
IGHV4-61	0.00215	1.16E-12	0.00132836	7.58E-05	0.02834375	3.08E-05	0.00012308
IGHV4-31	0.00431	1.78E-12	0.00110448	0.00424242	0.0015625	3.08E-05	7.69E-05
IGHV3-48	0.01940	2.36E-12	0.00032836	0.00040909	0.0001875	0.00096923	0.00096923
IGHV4-59	0.15302	2.77E-12	0.00011940	0.00013636	0.01929688	4.62E-05	0.00195385
IGHV3-11	0.00431	3.14E-12	0.00037313	0.00074242	0.00025	0.00015385	0.00306154
IGHV3-30-3	0	4.71E-11	0.00219403	0.01130303	0.00153125	0.00115385	1.54E-05

Lists the top ten scoring IGHV genes in the FPCA-based test for the B cell data. “Lit. Ab Freq.” lists the frequency for each of the V genes in the literature-curated dataset. “Combined” lists the *p* values for each of the V genes after using Fisher’s method to combine the *p* values from the FPCA-based test across all the patients. Patient 1–5 lists the *p* values for each of the individual patients from the FPCA-based test. Genes are sorted by combined *p* value

### Testing for a convergent V gene response

Recently there have been several studies that have reported independent AbRs showing signals of sequence convergence when challenged with a similar antigenic stimulus [15, 21, 41, 42]. This suggests that independent patients may use the same V genes to target similar antigens. Consistent with this, we found that V genes tend to have similar FPCA-based test scores across patients (Additional file 3: Figure S6). This suggests that the patients in our data are indeed using similar V genes to target TIV.

However, the sets of TIV-responding V genes were statistically inferred using time-series data, and were not shown to physically bind TIV. To validate these findings, we searched the literature for Abs that have been experimentally shown to bind either an influenza vaccine or the influenza virus itself. Since most publications do not provide sequence information for the Abs they find, our analysis is limited to the germline genes from which the Abs originated. Our search resulted in 464 Abs that have been shown to bind influenza vaccine or influenza virus (Additional file 3: Tables S2 and Additional file 4: Table S3). We then compared the TIV-responding V genes identified by our FPCA based test to the frequency of each V gene from our literature-curated dataset. Specifically, since each patient is approximately independent, we used Fisher’s method to combine FPCA-based *p* values across patients. This results in a single *p* value for each gene, where significance is increased if a gene is inferred to target TIV in multiple patients. Conversely, significance is diminished if a gene is heterogeneous across patients (Additional file 5: Table S4).

**Fig. 4:**
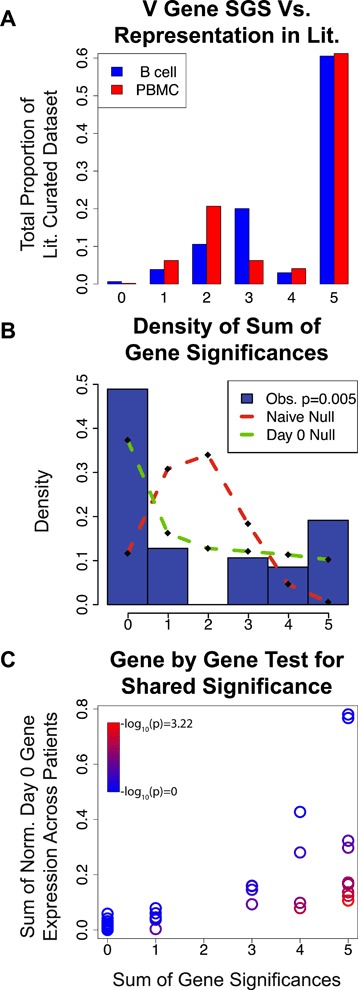
Identifying a convergent V gene usage signal across patients. The *x-axis* for all plots is the sum of gene significances (SGS) statistic, which is the number of patients for which a given Ab gene was found to be significant. **a** Comparing our results for IGHV to the literature. For each SGS bin, this shows the proportion of the Abs in the literature-curated data that have V genes belonging to this bin. Approximately 60 % of the influenza binding Abs in the literature-curated dataset were composed of V genes that had an SGS value of 5. **b** Comparing observed SGS to the null distribution. *Blue bars* are a *histogram* showing the observed proportion of IGKV genes from the PBMC data belonging to each SGS bin. *Red dashed line* shows the “naïve” null distribution of SGS if each patient were independent from one another (see “Methods”). The *green dashed line* shows the null distribution of SGS if the baseline similarity in gene expression at day 0 is taken into account. The *p* value in the legend shows the result of using a multinomial G test to compare the observed SGS distribution to that of the day 0 null. **c** Comparing the SGS value for each IGKV gene from the PBMC data to that of their respective null expectations. *Color* indicates the probability of the observed SGS under the null model (*p* value, see “Methods” for explanation of null model)

We found that these combined *p* values are correlated with IGHV gene frequency in the literature-curated dataset (Kendall’s Tau, B cell *p* = 3.115e-5, PBMC *p* = 2.502e-5). Moreover, we find that ~60 % of all Abs in our literature-curated dataset were composed of V genes that were inferred to be TIV-responding across all patients in our analysis (Fig. 4a). In particular, we find that the genes IGHV1-69 and IGHV3-7, which have been shown to consistently target influenza epitopes in several independent studies [41, 54–58] have the second and fourth lowest *p* values in the B cell data (Table 1), and first and second lowest *p* values in the PBMC data (Additional file 5: Table S4), respectively. One of the publications that contributed to our literature-curated dataset used a combinatorial phage display library to select for influenza-targeting Abs (Throsby et al. [54]). This is different from the in vivo selection process that occurs in humans and thus could introduce unknown bias in the Abs from this study. We removed the data from this study and saw no qualitative difference in the outcome (Additional file 3: Figure S7). Together, these data show that: (1) the V genes that we identify as TIV-responding with our pipeline are consistent with previous findings in the literature; and (2) that the patients from the Henn et al. dataset, as well as those from several other studies, tend to use similar V genes to target the influenza vaccine.

There are two reasonable explanations for this observation. The first is that some V genes have properties that make them naturally better at targeting TIV than others and are thus more likely to show a response across patients. The second is that patients tend to have similar V gene expression levels prior to vaccination, such that the Abs that are selected to respond to TIV tend to have similar V genes across patients simply due to this prior baseline similarity. We argue that this latter explanation has been underappreciated and thus merits further scrutiny.

Suppose V gene expression levels are correlated across patients, independent of any antigenic stimulus. If Ab lineages were randomly selected to respond to an antigenic stimulus (the null expectation), then we would expect to see similar V genes responding to said antigenic stimulus across patients purely due to the underlying correlation of V gene expression prior to inoculation. We tested for correlations in V gene expression levels prior to vaccination (day 0) and found that they are highly correlated across patients (Additional file 3: Figure S8). We therefore developed a statistical test that will take into account the underlying similarity in V gene expression prior to vaccination when determining if the patients in our data tend to use more similar sets of V genes to respond to TIV than would be expected by chance (see “Methods”). For each gene, we find the number of patients in which the gene is found to be significant by our FPCA test (referred to as Sum of Gene Significances, or SGS, Additional file 6: Tables S5 and Additional file 7: Table S6). We then compare the observed SGS distribution to a null. We found that the observed SGS distribution was significantly different than the null for IGKV from the PBMC dataset (multinomial G-test, *p* = 0.005; Fig. 4b; dashed green line vs. histogram) and we saw no evidence for convergent gene usage for other classes of V genes (Additional file 3: Figure S9). We then assessed the possibility that this convergent signal was driven by outlier genes that were deemed significant by the FPCA-based test, but do not have expression trajectories representative of a vaccine response (e.g. IGHV3-23 in patient 2, Fig. 3b). We performed a rather extensive outlier removal analysis to address this, where we removed these outliers in a variety of different ways (see Additional file 8: Appendix for a detailed description). In short, our convergent signal for IGKV was robust to all outlier removal approaches.

Given the mixed evidence for a global convergent signal in V gene response to TIV, we investigated each V gene individually (i.e. we test whether a given V gene was found to be TIV-responding in more patients than expected by chance). Similar to our global V gene analysis, we used the gene frequencies at day 0 to construct our null distribution (the null was solved in closed-form, as opposed to simulating; see “Methods”). We found that two V genes showed a significant convergent signal after Bonferroni correction for multiple testing. These were IGHV3-66 on the heavy chain and IGKV3-NL1 on the kappa light chain, using the PBMC data (Additional file 9: Tables S7 and Additional file 10: Table S8). In general, these significant V genes had a characteristic expression level trajectory of low expression prior to vaccination, and then increasing in expression post vaccination. This character of trajectory made it unlikely that the V genes would be selected to respond to the vaccine simply because they were abundant (or highly expressed) prior to vaccination, yet their increase in expression level after vaccination makes them likely candidates for responding to the vaccine.

To our knowledge, neither IGHV3-66 or IGKV3-NL1 have been reported to have shown a convergent response to TIV before and are absent from our literature-curated dataset. Conversely, the V genes IGHV1-69 and IGHV3-7—which have been reported in the past as showing a convergent signal when targeting TIV—are not significant in our test. This means that we cannot reject the possibility that these genes were found to be consistently targeting TIV simply due to their tendency to be highly expressed prior to vaccination. While it is possible that the reason for this initial high gene expression is because of prior convergences due to a similar antigenic history, it is also possible that these V genes are highly expressed independent of any antigenic history. We cannot differentiate between these two possibilities, so this baseline correlation must be corrected for.

Together, the results from our tests for a convergent signal in V gene usage show that some patients tend to use similar sets of V genes for particular gene classes and that a couple of these V genes stand out. While only a subset of our tests yielded a significant convergent signal, we found it notable that there was any convergent signal at all, given the strong baseline correlations across patients prior to vaccination.

### Testing for a convergent CDR3 response

**Fig. 5:**
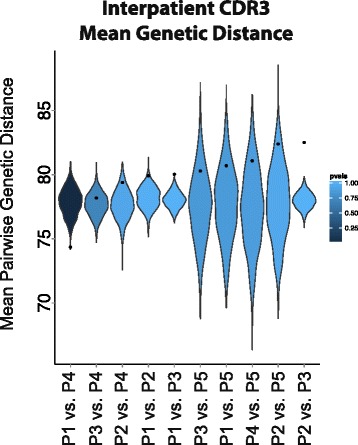
Testing for convergent CDR3 sequences across patients. *Black points* indicate the observed mean genetic distance between each pair of patients for the TIV-responding CDR3 sequences in the B cell data. *Violin plots* show the null distribution of mean pairwise distance values for each patient comparison (see “Methods” for how the null distribution was created). A point below the null distribution indicates convergent TIV-responding CDR3 sequences and above indicates divergent TIV-responding CDR3 sequences. Patient comparisons are sorted by observed mean pairwise genetic distance and distributions are colored by their empirical *p* value. P1 vs. P4 *p* value = 0.001

We hypothesized that if the patients within this dataset are capable of using similar sets of V genes to target the same vaccine, then they may use similar sets of CDR3 sequences to target TIV as well. To answer this, we began by again using the FPCA-based test on our CDR3 expression data to identify the putative TIV-responding CDR3 sequences. We were then left with a list of CDR3 sequences for each patient that appear to be responding to TIV. Our task was then to determine if these lists of TIV-responding CDR3s were more similar between patients than would be expected by chance (see “Methods”). We found that patients 1 and 4 seem to have converged on similar CDR3 sequences to target TIV, whereas patients 2 and 3, and patients 1 and 3 seem to have diverged (Fig. 5 and Additional file 3: Figure S10).

### Power calculations

In order to assess statistical power for our SGS based tests for convergence, we calculated power over a range of parameter values for both our global gene usage convergence test as well as our individual gene convergence test. See “Methods” for a detailed description of how this was done

To calculate power for our global gene usage test, we designed simulations where we can simulate a given number of truly convergent genes, as well as simulate additional patients. From this, we were able to determine how many truly convergent genes and how many patients are necessary to give sufficient power to differentiate from the null distribution. For example, one can imagine that if there were only five V genes that were truly convergent, then it might be difficult for the resulting distribution of SGS values to be statistically different from the null. However, if there were 50 patients in the dataset, then it would be unlikely for all 50 of these patients to “choose” the five convergent genes by chance and would allow for a statistical difference from the null. We ran these power simulations over a range of “number of patients,” and “number of convergent genes” parameter values (Additional file 3: Figure S11A). We found that in order to have strong power to reject the null, if there are five patients (as in our observed data), there must be greater than nine convergent genes.

We also calculated power for our individual gene convergence test over a range of parameter values. Here, the parameters that we varied were “number of patients” and “starting gene frequency” (frequency at day 0). In this case, if a gene were highly expressed at day 0 then it would be difficult for this gene to be statistically different from the null hypothesis, as it might be relatively easy for many patients to “choose” this gene to respond to TIV by chance. However, if there were 100 patients in the study, then it may be unlikely for this gene to be selected in all patients. We found that if there are five patients in the study, a gene must have a day 0 frequency lower than 0.06 in each of the patients in order to reliably reject the null hypothesis (Additional file 3: Figure S11B).

## Discussion

We have mined and characterized the global AbR response to TIV in five individuals from RNA-seq data. We find that individuals exhibit a heterogeneous response to TIV. Some of the patients showed characteristics of a monoclonal response, while others responded with much more of a polyclonal character. Interestingly, patient 1, who demonstrated characteristics of the most dramatic monoclonal response, was also the oldest patient (Additional file 3: Table S1). This is in line with previous work showing that older humans tend to have larger clonal expansions in their AbRs [12]. While all the individuals’ overall Ab expression increased markedly post vaccination, the timing and amplitude of this spike was variable. It is important to note that the patient with arguably the most dramatic Ab response to TIV also had a relatively early spike in overall Ab expression, which had almost completely subsided by day 7. This is the time-point that immunologists typically collect samples for vaccine response studies (see Galson et al. [2] and Table 1 for examples) and in this individual’s case the dramatic signal would have been all but lost if the traditional study design of pre- and post-vaccination time-points were used. This is consistent with the findings of Henn et al. [30] and further exemplifies the utility of study designs that emphasize dense, longitudinal sampling rather than cross-sectional sampling, as much of the signal would have been missed were there sparser sampling in the time-course. Further, as one decreases the number of time-points, it may become increasingly difficult to distinguish the signal from the noise, which would decrease the power to identify the elements responding to the stimulus.

While targeted sequencing of the Ab locus is unarguably the best way to illustrate the AbR, we, and others [35–37], have shown that a relatively simple bioinformatic pipeline can be implemented to characterize the AbR from RNA-seq data. This will hopefully provide investigators with the ability to leverage their RNA-seq data even further. Sequencing costs continue to plummet each year, however they still remain prohibitive for performing both targeted sequencing and RNA-seq for the average project budget. If one were interested in overall, population level statistics of the AbR, such as abundance or diversity, or if one were interested in finding/observing the Abs that are highly expressed in the AbR, we would argue that RNA-seq data are more than sufficient for these purposes. However, if one were interested in identifying rare Abs in the population, or needed full Ab sequences, then targeted sequencing of the Ab locus would be advised. In addition to prohibitive sequencing costs, targeted, deep-sequencing of the AbR remains a highly skilled method that involves a great deal of optimization, whereas RNA-seq has well vetted and broadly used protocols. In short, we hope that our method opens up the field of AbR analysis to a broader array of researchers.

The unique, densely sampled time-series dataset from Henn et al. [30] provided us with the ability to use functional data analysis methods to statistically identify putative TIV-responding V genes. We found V genes that were commonly TIV-responding across all patients in our dataset and that these commonly used V genes were also prevalent in influenza targeting Abs collected from the literature. This finding suggests that we have identified V genes that indeed function to target TIV. This also raises the intriguing possibility that some V genes are inherently better than others at targeting TIV, as independent patients seem to be selecting the same V genes to respond to the vaccine. If this were true, it would have interesting implications for the natural design and function of the diversity of genes in the AbR. For example, it could imply that instead of the different V genes providing the basis for an otherwise random exploration of sequence space when optimizing Abs, they could perhaps have evolved as “specialists” for particular classes of antigens, such that when an Ab is comprised of a particular V gene it is pushed in a particular direction of antigenic space.

As interesting as a convergent signal may be, one must exercise great caution when searching for one. If correlations between individuals exist prior to the selection event, then these correlations must be controlled for in any convergence test. For example, consider a V gene that is highly expressed in many individuals prior to vaccination and imagine that this V gene was found to be TIV-responding in many patients. As we have pointed out, one does not know if the reason that this V gene was found to be TIV-responding across patients is because it actually has a greater propensity to target TIV than other V genes or because it was selected randomly due to its high prevalence in the individuals. It is certainly possible that the highly expressed V genes have a greater propensity to target TIV. Indeed, it is possible that the reason they are highly expressed is because of prior vaccinations/antigenic exposure. However, we argue that it is equally possible that some Ab genes have a high endogenous expression level independent of any antigenic stimulus. Because of this, we do not have the statistical ability to de-convolute these two possibilities. Increasing the number of patients in these types of studies would help ameliorate this problem. However, as we show with our power calculations, one experiences diminishing returns in statistical power with adding patients to the study (Additional file 3: Figure S11). Alternatively, a synthetic AbR could be created that has a relatively even distribution of Ab elements and tested for activity against TIV (or other antigens as well).

Despite the strong correlations across patients in V gene expression levels prior to vaccination, we found statistically significant convergent signals in a subset of our tests. We observed global convergence for the IGKV genes, as well as convergence in the individual V genes, IGHV3-66 and IGKV3-NL1. As Dunand and Wilson [38] point out in their review, the V genes IGHV1-69 and IGHV3-7 have been implicated in convergent signals in a huge variety of contexts, including chronic lymphocytic leukemia [59, 60], Sjögren’s syndrome [61], and influenza [41, 55–58, 62–64] (for both IGHV1-69, and IGHV3-7), as well as human immunodeficiency virus [65–67] and hepatitus C virus [68] (for IGHV1-69 alone). Given that these genes were not significant in our convergence tests, and given the vast array of disparate antigens that these genes have been shown to “converge” towards, it seems that perhaps the simpler explanation may in fact be that these genes have high endogenous expression independent of any antigenic stimulus and are simply found to consistently respond to a diverse array of antigens by chance. This is a hypothesis that we feel deserves further scrutiny in future studies.

Our method for testing for a convergent signal in the AbR could be easily extendable to other systems. For example, this approach could be applied to meta-genomic microbiome data in order to identify taxa that are consistently responding to some stimulus. It could also be applied to infections in order to see which sequence characteristics of a given pathogenic population are consistently responding to (or resisting) a drug.

## Conclusions

We have shown that AbR information can be harvested from RNA-seq data, that a densely sampled time-series can be used to identify the Ab elements that are responding to a stimulus, and that patients tend to use similar Ab elements to target the same vaccine, albeit in certain cases.

## Additional files

Additional file 1: The PRISMA Statement. This accompanies the meta-analysis portion of this study (see “Methods”). (DOCX 29 kb) Additional file 2: The PRISMA flow diagram. Accompanies the PRISMA Statement. (PDF 331 kb) Additional file 3: Supplemental figures and supplemental Tables S1 and S2. Tables S3–8 are large so are instead included as separate CSV files (described below). Also includes the legends for all supplemental figures/tables that are cited in this manuscript (including Tables S3–8). (DOCX 2747 kb) Additional file 4: Table S3. Literature-curated dataset of flu-targeting Abs. (CSV 25 kb) Additional file 5: Table S4.
*P* values for FPCA-based test. (CSV 19 kb) Additional file 6: Table S5. TIV-responding genes for each patient, B cell data. (CSV 3 kb) Additional file 7: Table S6. TIV-responding genes for each patient, PBMC data. (CSV 3 kb) Additional file 8: The Appendix. Describes our outlier removal analysis. (DOCX 349 kb) Additional file 9: Table S7. Results for individual gene test for convergence, B cell data. (CSV 5 kb) Additional file 10: Table S8. Results for individual gene test for convergence, PBMC data. (CSV 4 kb)

## Abbreviations

Ab: Antibody
AbR: Antibody repertoire
CDR3: Complementarity determining region 3
D: Diversity antibody gene segment
FDA: Functional data analysis
FPCA: Functional principal components analysis
HAI: Hemagglutinin inhibition assay
IGH: Heavy chain of antibody
IGHV: Variable gene on heavy chain
IGK: Kappa light chain of antibody
IGKV: Variable gene on kappa chain
IGL: Lambda light chain of antibody
IGLV: Variable gene on lambda chain
J: Joining antibody gene segment
mAb: Monoclonal antibody
NGS: Next generation sequencing
PBMC: Peripheral blood mononuclear cell
PRISMA: Preferred reporting items for systematic reviews and meta-analyses
RSS: Residual sum of squares
SGS: Sum of gene significances
SHM: Somatic hyper mutation
TIV: Tri-valent influenza vaccine
V: Variable antibody gene segment
