# Supplementary material for: Statistical inference of a convergent antibody repertoire response to influenza vaccine
Source: Genome Med. 2016 Jun 3;8:60. doi: 10.1186/s13073-016-0314-z (PMC4891843; doi:10.1186/s13073-016-0314-z)
Supplement: Additional file 1: — The PRISMA Statement. This accompanies the meta-analysis portion of this study (see “Methods”). (DOCX 29 kb) [file 13073_2016_314_MOESM1_ESM.docx]

**Title:**

Meta-analysis of influenza targeting antibodies

**Abstract**

Sequence analysis of influenza targeting antibodies (Abs) is a well-studied field. There exist many reports of monoclonal Abs (mAbs) that have been isolated and found to bind various epitopes on the influenza virus. Further, there have been many qualitative reports of sequence similarities between influenza-targeting Abs identified in independent patients. Thus, we sought to generate a literature curated dataset of Abs that have been shown to target influenza in order to compare this set with the results of our analysis. We assessed a total of 14 manuscripts for inclusion in our meta-analysis and after a selection process arrived at seven studies [1–7] that met our criteria, and removed seven publications for various reasons [8–14]. The goal of this meta-analysis is to see if our findings of influenza targeting Abs agree with what has been previously reported in the literature. While we were unable to retrieve full sequence information from most of the studies included in this meta-analysis, we were able to gather the germline heavy chain genes that made up the reported influenza-targeting Abs as well as light chain genes when available. We see a general trend, where the Abs that have a strong TIV-targeting character in our analysis also are well represented in the literature curated dataset of influenza-targeting Abs. Particularly, we note that the heavy chain V genes, IGHV1-69 and IGHV3-7, which have been qualitatively shown to consistently target influenza [1, 2, 4, 5, 15, 16], are both well represented in the literature curated dataset as well as among the highest scorers in our analysis. These findings establish that the results of our analysis agree with the conglomerate of influenza-binding Abs that have been previously reported, and also suggest that certain V genes target influenza better than others.

**Methods**

**Rationale**

There exists much information on Abs that have been previously shown to target/bind influenza epitopes. However, the reported influenza-targeting Abs exist in many independent manuscripts and are not readily accessible as a single dataset. We sought to pool the influenza-targeting Ab information from all the manuscripts we could find and leverage this information in order to corroborate our results.

**Objectives**

We aim to create a database of the germline gene identity of previously reported influenza-targeting Abs, in order to test whether or not our independent findings agree with the contents of this database.

**Protocol and registration**

There is no review protocol registered for this meta-analysis.

**Eligibility criteria**

In order to be eligible for inclusion in our meta-analysis, the studies had to be reported in a peer-reviewed journal and had to be broadly on the topic of the effects of influenza stimulus on Abs in human subjects. They also had to provide (at a minimum) the germline gene identity of the heavy chain V gene (IGHV).

**Information sources**

We performed a literature search of all peer-reviewed journals up to the date of 14 June 2015.

**Search**

We employed commonly used search engines such as Google Scholar and PubMed to identify prospective studies for our meta-analysis.

**Study selection**

The studies included in this meta-analysis had to show that their reported Abs were capable of physically binding an influenza epitope. This epitope could be within an autologous or heterologous flu virus or reside on a flu vaccine. In order to establish physical interaction, the studies had to use an established experimental technique such as ELISA.

**Data collection process**

As the results of the selected studies were presented as tables in .pdf files, we manually extracted the germline gene identity of their reported influenza-targeting Abs.

**Data items**

The data collected from these manuscripts were the germline gene identity for each of the reported influenza-targeting Abs reported. That is, we gathered the identity of the IGHV, IGHD, IGHJ, IGLV, IGLJ, IGKV, and IGKJ genes wherever possible.

**Risk of bias in individual studies**The data from Throsby et al. [5] represents a bit of an outlier with respect to data collection methods. This is because the authors used a human derived AbR to generate a combinatorial phage display library of Abs. They then used this phage display library to select for Abs that bind to influenza epitopes. This is different from the in vivo selection process that occurs in humans, and thus could introduce a bias in the Abs from this study. Indeed, meaningful differences have been detected between the mAbs generated using an in vivo AbR to screen for mAbs vs. a synthetic AbR made using a combinatorial phage display library [17]. To address this, we conducted our analysis both with, and without the Throsby et al. data and found no qualitative difference in the outcome.

**Summary measures**

Each study that we used for our meta-analysis was summarized by taking the counts of each of the germline Ab genes that the authors reported to be influenza-targeting.

**Synthesis of results**

We combined the results of each study by simply summing the counts of each germline Ab gene for each study.

**Risk of bias across studies**

Different levels of screening for influenza-targeting Abs were done across studies. For example, some studies only reported the mAbs that were capable of binding epitopes from a broad array of influenza strains (i.e. broadly neutralizing Abs), while others reported mAbs that could bind any influenza epitope. The studies with less stringent criteria for reporting Abs, also had much more data. Thus, it is possible that “broadly neutralizing” Abs are slightly enriched in our data, however, their proportion in the meta-data is quite low.

**Additional analyses**

There were no additional analyses done on these data.

**Results**

**Study selection**

We scanned the literature for studies on the Ab response to an influenza epitope and, after a selection process, arrived at a total of seven studies [1–7] that met our criteria for inclusion in the meta-analysis. We began with a total of 14 manuscripts and assessed their eligibility [1–14]. From this original 14 we removed four studies [8–11] because they did not actually isolate any mAbs. We also removed one study [12] because it contained duplicate Ab data to another study [3] already in the set. Further, we removed one study [13] because it did not provide any germline gene Ab information for the influenza-targeting Ab that was isolated. Finally, we removed an additional study [14] because the authors used a protocol that involved injecting human derived B cells into humanized mice, and we were concerned that this non-human step would introduce undue bias in our final results. The final seven studies [1–7] gave us the germline Ab gene usage information for a total of 464 mAbs that had been shown to physically interact with an influenza epitope.

**Study characteristics**

The data that were extracted from each study were simply the germline Ab genes that composed the influenza-targeting Abs that were presented.

**Risk of bias within studies**

We conducted our analysis both including and excluding the Throsby et al. [5] data and found no qualitative differences in our final results or conclusion regarding this analysis.

**Synthesis of results**

We compiled the gene usage information from each of the studies for IGHV and compared this to the TIV-targeting genes that we identified in our analysis. We found that the *p* values for each IGHV gene from our FPCA based analysis are significantly correlated with the IGHV gene frequency from this meta-analysis dataset (Kendall’s tau *p* value for B cell data = 3.115e-5; PBMC data = 2.502e-5). We also did this while excluding the Throsby et al. data and found similar results (Kendall’s tau *p* value for B cell data = 5.591e-5; PBMC data = 4.519e-5).

**Additional analysis**

No additional analyses were done.

**Discussion**

**Summary of evidence**

Together, this meta-analysis, when compared to our results, shows that: (1) our results agree with what has been previously found; and (2) that influenza targeting Abs tend to use similar IGHV genes.

**Limitations**

While much work has been done on influenza immunology, there are still relatively few studies that systematically search for influenza-targeting Abs. This meta-analysis would benefit from a larger number of studies included. In addition, a more in-depth analysis would be possible if the full Ab sequence information (at the nucleotide level) were published in these studies, as opposed to only the germline gene information.

**Conclusions**

See “Summary of evidence.”

**Funding**

This study was supported by grants from the National Institutes of Health (1R01HG007644 and 1R21HG007233), the Center For Aids Research (CFAR) at UCSF, and an Alfred P. Sloan Foundation Fellowship to RDH, as well as a Genentech Predoctoral Fellowship and the Silvio Canonica Scholarship from the Swiss Benevolent Society to NBS.

**References**

1. Wrammert J, Koutsonanos D, Li G-M, Edupuganti S, Sui J, Morrissey M, et al. Broadly cross-reactive antibodies dominate the human B cell response against 2009 pandemic H1N1 influenza virus infection. J Exp Med. 2011;208:181–93.

2. Krause JC, Tsibane T, Tumpey TM, Huffman CJ, Briney BS, Smith SA, et al. Epitope-specific human influenza antibody repertoires diversify by B cell intraclonal sequence divergence and interclonal convergence. J Immunol. 2011;187:3704–11.

3. Moody MA, Zhang R, Walter EB, Woods CW, Ginsburg GS, McClain MT, et al. H3N2 influenza infection elicits more cross-reactive and less clonally expanded anti-hemagglutinin antibodies than influenza vaccination. PLoS One. 2011;6:e25797.

4. Corti D, Suguitan AL, Pinna D, Silacci C, Fernandez-Rodriguez BM, Vanzetta F, et al. Heterosubtypic neutralizing antibodies are produced by individuals immunized with a seasonal influenza vaccine. J Clin Invest. 2010;120:1663–73.

5. Throsby M, van den Brink E, Jongeneelen M, Poon LLM, Alard P, Cornelissen L, et al. Heterosubtypic neutralizing monoclonal antibodies cross-protective against H5N1 and H1N1 recovered from human IgM+ memory B cells. PLoS One. 2008;3:e3942.

6. Human monoclonal antibodies to pandemic 1957 H2N2 and pandemic 1968 H3N2 influenza viruses. http://jvi.asm.org.

7. Yu X, Tsibane T, McGraw PA, House FS, Keefer CJ, Hicar MD, et al. Neutralizing antibodies derived from the B cells of 1918 influenza pandemic survivors. Nature. 2008;455:532–6.

8. Vollmers C, Sit RV, Weinstein JA, Dekker CL, Quake SR. Genetic measurement of memory B-cell recall using antibody repertoire sequencing. Proc Natl Acad Sci. 2013;110:13463–8.

9. Dekosky BJ, Ippolito GC, Deschner RP, Lavinder JJ, Wine Y, Rawlings BM, et al. High-throughput sequencing of the paired human immunoglobulin heavy and light chain repertoire. Nat Biotechnol. 2013;31:166–9.

10. Jiang N, He J, Weinstein JA, Penland L, Sasaki S, He X-S, et al. Lineage structure of the human antibody repertoire in response to influenza vaccination. Sci Transl Med. 2013;5:171ra19–171ra19.

11. Hoek KL, Samir P, Howard LM, Niu X, Prasad N, Galassie A, et al. A cell-based systems biology assessment of human blood to monitor immune responses after influenza vaccination. PLoS One. 2015;10:e0118528.

12. Jackson KJL, Liu Y, Roskin KM, Glanville J, Hoh RA, Seo K, et al. Human responses to influenza vaccination show seroconversion signatures and convergent antibody rearrangements. Cell Host Microbe. 2014;16:105–14.

13. Corti D, Voss J, Gamblin SJ, Codoni G, Macagno A, Jarrossay D, et al. A neutralizing antibody selected from plasma cells that binds to group 1 and group 2 influenza A hemagglutinins. Science. 2011;333:850–6.

14. Nakamura G, Chai N, Park S, Chiang N, Lin Z, Chiu H, et al. An in vivo human-plasmablast enrichment technique allows rapid identification of therapeutic influenza A antibodies. Cell Host Microbe. 2013;14:93–103.

15. Ekiert DC, Bhabha G, Elsliger M-A, Friesen RHE, Jongeneelen M, Throsby M, et al. Antibody recognition of a highly conserved influenza virus epitope. Science. 2009;324:246–51.

16. Sui J, Hwang WC, Perez S, Wei G, Aird D, Chen L, et al. Structural and functional bases for broad-spectrum neutralization of avian and human influenza A viruses. Nat Struct Mol Biol. 2009;16:265–73.

17. Saggy I, Wine Y, Shefet-Carasso L, Nahary L, Georgiou G, Benhar I. Antibody isolation from immunized animals: comparison of phage display and antibody discovery via V gene repertoire mining. Protein Eng Des Sel. 2012;25:539–49.
