# Supplementary figures and images for: Statistical inference of a convergent antibody repertoire response to influenza vaccine
Source: Genome Med. 2016 Jun 3;8:60. doi: 10.1186/s13073-016-0314-z (PMC4891843; doi:10.1186/s13073-016-0314-z)

Identification

Screening

Eligibility

Included

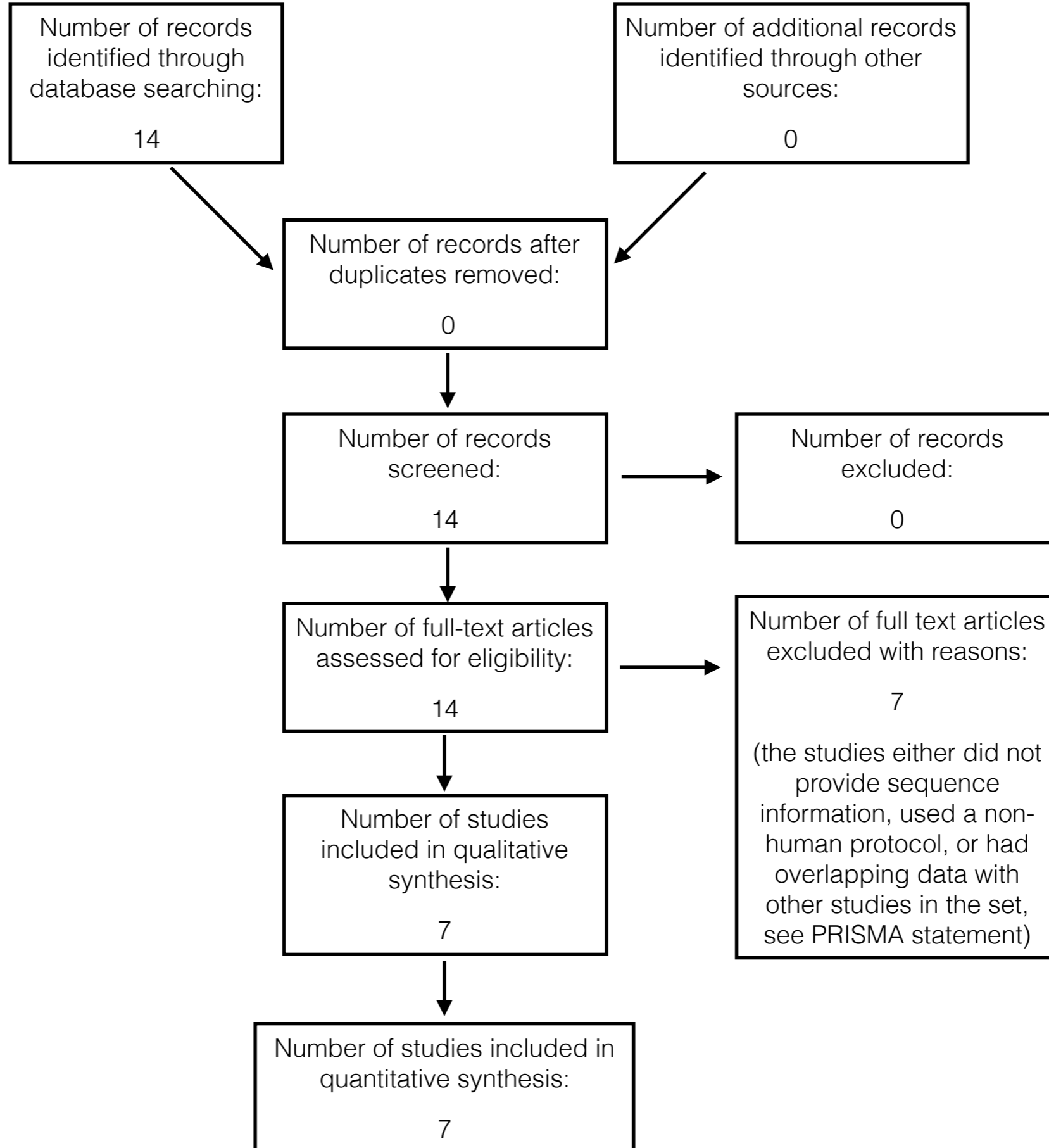

Supplement: Additional file 2: — The PRISMA flow diagram. Accompanies the PRISMA Statement. (PDF 331 kb) [file 13073_2016_314_MOESM2_ESM.pdf]
