# Supplementary material for: Statistical inference of a convergent antibody repertoire response to influenza vaccine
Source: Genome Med. 2016 Jun 3;8:60. doi: 10.1186/s13073-016-0314-z (PMC4891843; doi:10.1186/s13073-016-0314-z)
Supplement: Additional file 3: — Supplemental figures and supplemental Tables S1 and S2. Tables S3–8 are large so are instead included as separate CSV files (described below). Also includes the legends for all supplemental figures/tables that are cited in this manuscript (including Tables S3–8). (DOCX 2747 kb) [file 13073_2016_314_MOESM3_ESM.docx]

**Supplemental Figure/Table legends**

**Figure S1.** Study design. *Schematic representation* of the vaccination study. There are five patients and each patient is given the same vaccine. Whole blood is drawn immediately prior to vaccination and each day for 10 days post vaccination (11 time-points total). Each patient/time-point sample is dived into three different sample types: B cells, PBMCs, and serum. The B cells and PBMCs are used for RNA-seq. The serum is used for immunological assays. This figure shows the same information as Figure S1 from Henn et al. [29].

*Patient avatars in no way reflect actual patients.

**Figure S2.** Correlation between B cell and PBMC AbR data. **A** *Scatter plot* showing the correlation between overall Ab expression in the B cell data to that of the PBMC data for each patient/time-point. *Points* are colored by time-point. **B** Same as (**A**) but showing correlation of mean pairwise diversity of CDR3 sequences between the B cell and PBMC data. See “Methods” for how the diversity statistic was calculated.

**Figure S3.** Comparing the B cell AbR to the PBMC AbR. *Density plots* showing the distribution of genetic distance values for randomly selected CDR3 sequences. Randomly selected sequences were matched by time-point. *Red lines* show the resulting distribution when comparing CDR3 sequences within the B cell data, *blue lines* show the same when using the PBMC data, and *purple lines* show the genetic distance distribution when comparing between datasets. Subtitle below the plots list the Mann–Whitney U *p* values when comparing the *red* and the *blue* distributions to the *purple*. **A** Patient 1. **B** Patient 2. **C** Patient 3. **D** Patient 4. **E** Patient 5.

**Figure S4.** Lambda and kappa Ab gene’s expression over time. *Stacked area charts* showing the cumulative as well as individual Ab gene expression over time for both the lambda and kappa chains. (**A**, **B**) *Stacked area charts* for IGLV genes from the B cell and PBMC data, respectively. (**C**, **D**) Same as (**A**) and (**B**) but for the IGKV genes. All distinct colors for plots of IGLV genes correspond to the same genes (i.e. colors are comparable across patients and sample types). The same is true for plots of IGKV genes.

**Figure S5.** CDR3 expression over time. *Stacked area charts* showing the cumulative as well as individual expression level for the 100 most frequent CDR3 sequences in the data. **A** B cell data. **B** PBMC data.


**Figure S6.** Correlation of TIV-responding V genes across patients. Comparison of FPCA based test *p* values between all pairwise patients, for each V gene. For each pairwise patient comparison, these plots show the correlation of the *p* values from the FPCA based test for each of the genes. Points are colored by patient comparison. Correlation *p* value (Kendall’s Tau) is listed in the title of each plot. **A** *Scatter plots* for IGHV, IGKV, and IGLV genes’ *p* values for the B cell data. **B** Same as (**A**) but for the PBMC data.

**Figure S7.** Comparison of SGS to literature-curated dataset, excluding Throsby et al. For each SGS bin, this shows the proportion of the Abs in the literature-curated data that have V genes belonging to this bin. The genes that were significant in all five patients represented the largest proportion of the genes shown to be influenza binding in the literature.

**Figure S8.** Baseline correlation of Ab gene expression at day 0. Comparison of day 0 expression level between all pairwise patients, for each TIV-responding V gene. For each pairwise patient comparison, these plots show the correlation of the day 0 expression level for each of the TIV-responding V genes. **A** Scatter plots for IGHV, IGKV, and IGLV TIV-responding genes’ day 0 expression level for the B cell data. **B** Same as (**A**) but for the PBMC data.

**Figure S9.** Testing for a global gene usage convergent signal. Comparing observed SGS to the null distribution. *Blue bars* are histograms showing the observed proportion of V genes belonging to each SGS bin. *Red dashed lines* show the null distribution of SGS if each patient were independent from one another. *Green dashed lines* show the null distribution of SGS if the baseline similarity in gene expression at day 0 is taken into account. The *p* values in the legends show the result of using a multinomial G test to compare the observed SGS distributions to that of the day 0 nulls. **A** *Histograms* of the IGHV, IGKV, and IGLV genes, for the B cell data. **B** Same as (**A**) but for the PBMC data.

**Figure S10.** Testing for convergent CDR3 sequences across patients. *Violin plots* showing the null distribution of mean pairwise distance values for each pairwise patient comparison (see “Methods” for how null distribution was created), for the PBMC data. *Points* indicate the observed mean genetic distance for the TIV-responding CDR3 sequences between the patient comparison. A point below the null distribution indicates convergent TIV-responding CDR3 sequences and above indicates divergent TIV-responding CDR3 sequences. Distributions are *colored* by *p* value with respect to the observed value. Patient 5 is absent because he had no statistically significant TIV-responding CDR3 sequences in the PBMC data.

**Figure S11.** Power calculations. Illustrates the statistical power over a range of parameter values for each of the gene usage convergence tests. **A** Power over a range of simulated patients and simulated convergent genes for the global gene usage convergence test. Up to 50 convergent genes were simulated but all sets of simulations with greater than 10 convergent genes yielded a power of 1. **B** Power over a range of patients and day 0 gene frequencies for the individual gene convergence test. Day 0 gene frequency was set to be equal across all patients for each power calculation. Tests with a starting gene frequency of up to 1.0 were run, however, every test with a starting gene frequency greater than 0.4 had a power of 0.0. IGHV expression data from the B cell dataset was used for the simulations/calculations in both (**A**) and (**B**).

Top portion of table lists the demographics and vaccination history for each of the patients. If a patient has an “X” under one of the vaccinations it means that that individual received the vaccine; a blank means that they did not. The lower portion of the table lists the strains used in each of the vaccines. Entries with thick borders highlight the strains that were used in the study TIV (Seasonal 2010). This table shows the same information as Table S1 from Henn et al. [29].

Lists all the publications that contributed to the literature-curated dataset. An “X” under a given class of Ab genes means that that gene class was reported in the publication. If an entry is blank, then the gene class was absent. “Number Abs Retrieved” lists the total number of monoclonal Abs that each publication contributed to the literature-curated dataset.

**Table S3. Literature-curated dataset of flu-targeting Abs.** Lists the germline gene identity for each of the Abs in the compiled literature-curated dataset. If an entry has an “NA” then that gene class was not reported in the publication. If the genes for one of the light chains are left blank, then that means that the given Ab was not composed of that light chain. The Wrammert (2011) study did not amplify lambda genes, and thus none of the Abs from this study were composed of the lambda light chain.

**Table S4. *P* values for FPCA-based test.** Reports the *p* values for each of the detected V genes from the FPCA-based test to identify those that are TIV-responding. “Lit. Ab Freq.” lists the frequency of each V gene in the literature-curated dataset. “Combined All,” “Combined B cell,” and “Combined PBMC” list the *p* values when Fisher’s method is used to combine the *p* values across all patient/sample types, across patients using B cell data and across patients using PBMC data, respectively. B cell Patients 1–5 lists the *p* values for each V gene for individual patients using the B cell data, and PBMC Patients 1–5 does the same using PBMC data.

**Table S5. TIV-responding genes for each patient, B cell data.** Reports the TIV-responding status for each of the V genes detected in our study, for each of the patients. If a given V gene has a 0 under a given patient, then that V gene was not deemed to be TIV-responding by our FPCA-based test for that patient, whereas if there is a 1, then the V gene was TIV-responding. SGS is the “Sum of Gene Significances” statistic and is sum across each row. V genes are sorted by their SGS value.

**Table S6. TIV-responding genes for each patient, PBMC data.** Same as Table S5, except using the PBMC data. See legend of Table S5 for description of data.

**Table S7. Results for individual gene test for convergence, B cell data.** Lists the *p* values for each V gene’s test for convergence, using the B cell data. “Obs. SGS” gives the observed SGS statistic for each gene. “Mode Null Dstrb. SGS” lists the mode of the calculated null distribution for the SGS statistic. “Sum of Day 0 Freqs.” gives the sum of the frequencies at day 0 across patients for a given gene. This is meant to provide a summary statistic to describe the level of expression (across patients) for each gene at day 0.

**Table S8. Results for individual gene test for convergence, PBMC data.** Same as Table S7, except using the PBMC data. See legend of Table S7 for description of data.
