# Supplementary material for: Statistical inference of a convergent antibody repertoire response to influenza vaccine
Source: Genome Med. 2016 Jun 3;8:60. doi: 10.1186/s13073-016-0314-z (PMC4891843; doi:10.1186/s13073-016-0314-z)
Supplement: Additional file 8: — The Appendix. Describes our outlier removal analysis. (DOCX 349 kb) [file 13073_2016_314_MOESM8_ESM.docx]

**Appendix**

We performed a somewhat extensive outlier removal analysis to control for the possibility that V genes that were deemed significant by the FPCA-based test [1], yet do not show expression trajectories representative of a vaccine response, were driving our convergent signals. We looked through our data and found that there is a small set of instances where the V gene expression functions fit this description. Here, we seek to identify the reason for this strange behavior in the test and go on to remove these outlier V genes to see if they have any effect on the convergent signal that we originally observed. We restrict our analysis to V gene Kappa in the PBMC data, as this was the only case where we saw a significant convergent signal.

Figure 1. *Plots of each gene expression function that were found to be significant by the FPCA-based test, for each patient. From left to right: patient 1, 2, 3, 4, and 5*

Figure 1 shows the expression trajectories for each Kappa V gene that was identified as significant by the FPCA-based test, for each patient. One can see that the majority of these V genes exhibit expression trajectories characteristic of a vaccine response, while a small subset of the V genes exhibits a behavior of simply decreasing in expression. These V genes were exclusively identified in patient 2. We found that the first eigenfunction in patient 2 explained only ~70 % of the variance in the data (as compared to >90 % of variance explained by the first eigenfunction in most other patients). The FPCA-based test works by taking the top set of eigenfunctions that cumulatively explain at least 90 % of the variance in the data in order to characterize the prevailing signal. For patient 2, the first and second eigenfunctions together explained ~98 % of the variance, so these were the two that were used for this individual. For a given gene, the FPCA-based test then fits a linear combination of these two eigenfunctions such that it is the best fit to the observed data for that gene. It then asks if this linear combination of the top two eigenfunctions is a better fit to the data than just a constant function that is equal to the mean expression.

Figure 2. *Plots of the first and second eigenfunctions (left and right, respectively) for patient 2.*

Figure 2 shows the first and second eigenfunctions for patient 2. One interpretation of these two eigenfunctions is that the first is capturing the signal from the V genes that are increasing in expression as a response to the vaccine and the second is capturing the signal from the V genes that are necessarily decreasing in expression as the TIV-responders rise in the sample. It so happens that certain linear combinations of the top two eigenfuctions in patient 2 can yield expression trajectories that do not track well with a vaccine response. We found that this only occurs for patient 2. In order to prevent this from happening, we restricted the FPCA-based test to only consider the first eigenfunction to characterize the prevailing signal in the data. We did this by setting the “FVE” threshold in the FPCA-based test to 0.70. That is, we made it so the FPCA-based test now takes the first set of eigenfunctions that cumulatively explain just 70 % of the variance in the data (as opposed to 90 % previously) to characterize the overall signal in the data. The effect of this is to force the test to only consider the first eigenfunction in its goodness of fit test, for each of the patients. Thus, eliminating the possibility of creating linear combinations of the first and second eigenfunctions that can yield strange expression trajectories. In general, this is ill-advised, as one is effectively missing out on up to 30 % of the signal in the data, which would cause the test to be underpowered. However, we reasoned that if we were to run the test in this fashion, and then still find a convergent signal in our downstream convergence test, then we can be relatively comfortable with our results. Indeed, we ran the FPCA-based test with FVE set to 0.70 and then ran our gene usage convergence test on the results, and found that our convergent signal remained (*p* = 0.007).

While this is a drastic and broad way to singlehandedly remove all the outliers in the data (likely with significant collateral damage on statistical power), we next sought to be more precise and observe the effects of removing one outlier at a time. To do this we first created a list of genes that were found to be significant by the FPCA-based test for patient 2, yet exhibited expression trajectories that visually did not seem representative of a vaccine response.

Figure 3. *Plots of observed gene expression and estimated gene expression functions for each outlier gene identified. Black points are the observed gene expression values, and red lines are the fitted curves to these data. Genes are sorted by their associated p value from the FPCA based test, in descending order. V gene names from left to right: IGKV1-13, IGKV1-16, IGKV1-9, IGKV2-29, IGKV1D-8, IGKV1-5.*

Figure 3 shows each of the identified expression trajectories. We then sorted these genes by their corresponding *p* values from the test, in descending order. Next, we set different numbers of the genes in this list to no longer be significant by the FPCA-based test and then observed the effect on our gene usage convergence test. More specifically, we started by making the first gene in this list no longer significant by the FPCA-based test and observed the results in our convergence test. We then made the first two genes in this list no longer significant and observed the results on the convergence test. Followed by making the first three genes to be no longer significant and observed the effects on the convergent signal, and repeated this until all the outlier genes were removed from the convergence analysis.

Figure 4. *P value from the global gene usage convergence test after each round of outlier gene removal.*

As Figure 4 shows, we found that our convergent signal is robust to the sequential removal of these outliers.

Figure 5. *Plots of observed gene expression and estimated gene expression functions for each of the remaining significant V genes, after removal of all outliers. Black points are the observed gene expression values, and red lines are the fitted curves to these data. V gene names from left to right, top to bottom: IGKV1-6, IGKV1-27, IGKV1-33, IGKV1D-17, IGKV2-24, IGKV2D-28, IGKV3/OR2-268, IGKV3-7, IGKV3-11, IGKV3-20, IGKV3-NL1, IGKV3D-15, IGKV4-1, IGKV2D-30.*

Figure 5 shows that the remainder of gene expression trajectories in patient 2, seem to fit a canonical vaccine response.

We conclude that the convergent signal that we detect for V gene Kappa in the PBMC data is a signal that is generated from the V genes that appear to be responding to the vaccine, and while the outlier genes that do not show gene expression trajectories characteristic of a vaccine response likely add noise to our test, they are not responsible for our original convergent signal.

Another aspect of our results that rely on the output of the FPCA-based test is our test for convergent CDR3 response. Here we found that patient 1 and patient 4 in the B cell data had more similar TIV-responding CDR3 sequences than would have been expected by chance. We now wish to assess the possibility that this convergent signal was generated by CDR3 sequences that were found to be significant by the FPCA-based test, yet do not exhibit characteristic vaccine responding expression curves.

Figure 6. *Plots of the first eigenfunctions in the CDR3 sequence expression data for patients 1 (left) and 4 (right). The proportion of variance explained: 99.32 % (left) and 97.52 % (right).*

We found that the first eigenfunctions in patient 1 and patient 4 explained 99.32 % and 97.52 % of the variance in the data, respectively (Figure 6). This means that the FPCA-based test would solely use the first eigenfunction in each of these patients for its goodness of fit test. Thus, a CDR3 sequence expression trajectory would have to match the shape of its corresponding first eigenfunction, and *only* this eigenfunction, to be deemed significant in either of these patients. While it would be too time-consuming to scan through each significant CDR3 sequence expression function to visually check that they are all exhibiting a characteristic vaccine response, it is extremely unlikely that any CDR3 expression function that does not recapitulate the general shape of its corresponding first eigenfunction would be found to be significant for these patients. Because of this, we conclude that the convergent signal observed between the CDR3 sequences of patient 1 and patient 4 in the B cell data is not influenced by CDR3 sequences falsely identified to be TIV-responding by the FPCA-based test.

**References**

1. Wu S, Wu H. More powerful significant testing for time course gene expression data using functional principal component analysis approaches. BMC Bioinformatics. 2013;14:6.
